# Self-Related Views of Aging After Age 50: The Long-Term Effects of Poor Health in Childhood

**DOI:** 10.1093/geroni/igaa057.1045

**Published:** 2020-12-16

**Authors:** Jacqui Smith, Marina Larkina

**Affiliations:** University of Michigan, Ann Arbor, Michigan, United States

## Abstract

Age stereotypes and expectations about one’s own aging commence in childhood but most research focuses on predictive associations with midlife health behaviors, later-life chronic conditions, and longevity. Surprisingly little is known about the role of poor childhood health in these associations. This study aims to fill this gap. Using data from the Health and Retirement Study (HRS: N = 5807; aged 50-98), we investigated whether diagnosed chronic illness before age 16 and self-rated childhood health predict late-life self-perceptions of aging (SPA) and subjective age discrepancy (AD). We conducted multivariate multiple regressions to determine the joint and unique effects of childhood health. Models included controls for current health (functional limitations), memory status, and demographic covariates (age, gender, race/ethnicity, marital status, and education). Multivariate tests (Pillai’s trace) revealed that both childhood health indicators were significant predictors. Over and above all covariates and the covariation of the two views of one’s own aging, univariate models showed that the number of childhood diagnoses was significant predictor of AD (p < .007) but not for SPA. In contrast, self-rated childhood health was a significant predictor of SPA (p < .001) but not for AD. This study provides new insight into precursors of self-evaluations of aging. Whereas childhood diagnoses of chronic illness attenuated the extent that individuals felt younger than their actual age, ratings of poor childhood health enhanced negative SPA. The non-normative experience of poor health in early life is a lifelong foundation for both late life beliefs and health.

